# Maxillofacial fractures in a budding teaching hospital: a study of pattern of presentation and care

**DOI:** 10.11604/pamj.2017.26.218.11621

**Published:** 2017-04-24

**Authors:** Obitade Sunday Obimakinde, Kolawole Olubunmi Ogundipe, Taopheeq Bamidele Rabiu, Victoria Nwebuni Okoje

**Affiliations:** 1 Ekiti State University Teaching Hospital, Ado-Ekiti, Nigeria; 2 Lautech Teaching Hospital, Osogbo, Nigeria; 3 University College Hospital, Ibadan, Nigeria

**Keywords:** Maxillofacial fractures, aetiology, pattern, altered consciousness, concomitant injuries

## Abstract

**Introduction:**

Previous reports indicated that there is geographic and sociodemographic variation in the epidemiology of maxillofacial fractures. Audit of maxillofacial injuries managed at any institution is therefore necessary to understand the trends and proffer strategies for prevention. We therefore embarked on this study to determine the pattern of maxillofacial fractures and concomitant injuries in our institution.

**Methods:**

We carried out a retrospective review of information on demography, aetiology and type of maxillofacial fracture, patients' status, type of crash, level of consciousness and concomitant injuries. The data collected was analysed with SPSS Version 20.

**Results:**

A total of 233 patients aged 2 to 66 years were reviewed. A higher male preponderance (M:F 3.4:1) was observed. Road traffic crashes (RTC) accounted for 78.5% of injuries. Motorcycle related crashes were responsible for 69.4% of RTC and 54.5% of all fractures. Fracture of the mandible (63.2% n=172) was the most predominant skeletal injury and the body (25% n=43) was the most common site of fracture while the zygoma (29%) was predominantly affected in the midface. Ninety three patients (40%) suffered loss of consciousness. The relationship between aetiology of injuries and consciousness level of the patients was statistically significant (p=0.001). Of the 43 patients who had concomitant injuries, craniocerebral affectation (60.5%) was the commonest.

**Conclusion:**

RTC remains the major aetiology of maxillofacial fractures. The mandible was mostly affected and nearly half of the patients have associated loss of consciousness. There is need for continual advocacy and enforcement of laws on preventive measures among road users.

## Introduction

Skeletal injuries of the face constitute a substantial proportion of trauma globally [1-4]. Oral and maxillofacial injuries can pose considerable long term functional, aesthetic and psychological complications [3, 5]. Such injuries constitute huge economic burden due to treatment costs, disabilities and man hour loss occasioned by hospital stay [6-8]. The epidemiology of maxillofacial fractures in different populations varies in type, severity and aetiology depending on the socio economic risk factors and cultural differences [9-12]. Brown and Cowpe [13] listed the aetiologic factors as road traffic crashes (RTC), falls, sports, industrial accidents, gunshots and assault. Some authors have also reported cases of animal attacks [14, 15]. Although some studies indicated that assault is the leading cause of maxillofacial injuries in United States of America and most European nations [16-18], however, RTC remain the major aetiology in most developing countries including Nigeria [6, 19-21]. The high prevalence of RTC in developing countries has been linked to heavy reliance on road as the major mode of transportation [22, 23]. Furthermore a sizeable number of unemployed youths take up jobs such as hawking and commercial motorcycling which predisposes them to RTC [7]. In 2010, World Health Organization estimated that 20-50 million victims of RTC suffer various forms of injuries and disabilities and this figure was projected to rise by 65% by the year 2020 [24]. Periodic audit of facial injuries helps policy makers to develop new strategies for prevention and to assess the proficiency of existing laws on road safety regulations and social habit of different populations [6-10]. There is no previous published work on this subject from our institution which hitherto was a secondary health care facility prior to its upgrade to a tertiary centre in 2008. Thus, the present study was designed to analyse the pattern of maxillofacial fractures and associated concomitant injuries encountered in our institution for comparison with reports from other parts of the globe.

## Methods

This study was carried out at Ekiti State University Teaching Hospital. It involved a retrospective review of records of patients who were referred for management of facial bone fractures between January 2010 and December 2014. The patients presented either through accident and emergency ward or maxillofacial surgery clinic of our hospital. Our institution serves as a major referral centre for Ekiti state and adjoining states in southwestern and northcentral part of Nigeria. Ethical approval for the study was obtained from our institution's research and ethics board. Information on demography, aetiology and type of fracture, examination findings and radiologic diagnosis were accessed from patients' individual proforma. Level of consciousness was determined using the Glasgow Coma Scale [GCS]. Diagnosis of maxillofacial fractures was achieved through clinical and radiologic evaluation. All patients had plain radiographs for radiologic evaluation. Additionally, Computarised Tomography Scan [CT scan] was done for those who could afford it. Type of treatment offered and concomitant injuries were also documented. Data obtained were summarised in frequency tables and analysed using SPSS statistical software package version 20. Descriptive statistics and Chi square test were carried out and the level of significance was set as p<0-05.

## Results

**Figure 1:**
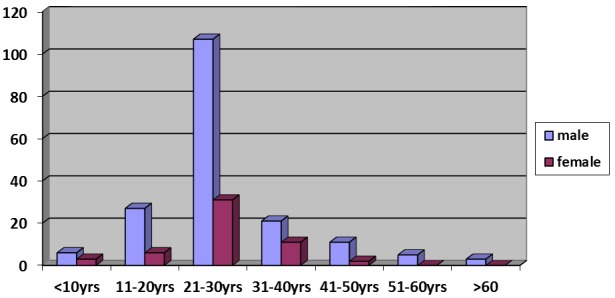
Revealed that the peak age incidence for oral and maxillofacial injury in this study is 21-30 years closely followed by 31-40 years. The least affected age groups were those above 50 years

A total of 233 patients with maxillofacial fracture were seen during the study period. Patients' demography revealed a male preponderance of 180 to 53 (M:F 3.4:1) while the mean age was 29.6±11.78 (age range: 2-66 years). There was a comparatively higher prevalence of patients between 20 and 30 years of age Figure 1. Analysis of the aetiology of injuries Table 1 revealed that RTC (78.5%, n=183) accounted for the majority followed by assault (19.7%). Motorcycle (MC) related crashes (n=127) was responsible for 69.4% of RTC and 54.5% of all cases of maxillofacial fractures studied. On the other hand, motor vehicle (MV) crashes was responsible for 24% of all injuries in this study. Thus motorcycle crashes was more than twice as common as those of motor vehicle (ratio 2.3:1).Table 2 showed that motorcycle riders were more involved in RTC related injuries than the pillion passengers while the contrary is the case with motor vehicle where most of the victims were passengers (p=0.006). Patients' record showed that only 19.7% (n=25) of victims of motorcycle accidents used protective/ crash helmet at the time of injury. Two hundred and seventy two fractures were seen in 233 patients with skeletal injuries giving an average of 1.2 fractures per patient. Table 3 showed that the mandible was the most commonly fractured facial bone (63.2%). The commonest site was the body (25%) in the mandible and Zygomatic bone (29%) in the mid face. Isolated dentoalveolar segment fractures accounted for 22.8% of all skeletal injuries seen in this study (n=62). Ninety three patients (40%) of the patients had associated altered state of consciousness (Glasgow coma scale less than 15) as at the time of clinical evaluation Table 4. Sixty seven (72.0%) regained full consciousness within 24 hours of admission. A greater proportion of patients who had altered consciousness were involved in motorcycle related injuries. Further analysis revealed a statistically significant relationship between aetiology of injury and altered consciousness (p=0.001). There was also a statistically significant relationship between fracture site [midface or mandible] and level of consciousness (p=0.003). Concomitant injuries occurred in 13.5% (n=43) of the patients and craniocerebral injury [skull fracture and cerebral concussion] accounted for 60.5% of such Table 5. Of the 272 fractures reviewed, 195 (71.7%) received treatment and majority (64.6%) were treated conservatively, predominantly with maxillo-mandibular fixation (MMF) while the remaining sixty nine patients (35.4%) had open reduction and internal fixation Table 6 .

**Table 1:** Aetiology of oral and maxillofacial injuries by gender

Aetiology	sex		total (%)
	Male	female	
RTC (MVA)	41	15	56 (24)
Motorcycle	99	28	127 (54.5)
Falls	2	0	2 (0.9)
Assault	36	10	46 (19.7)
Sports	2	0	2 (0.9)
Total	180	53	233 (100)

**Table 2:** Status of patients involved in RTC

Agent of RTC	Driver/rider (%)	Passenger (%)	Pedestrian (%)	Total (%)
Motor vehicle	10 (17.9)	42 (75)	4 (7.1)	56 (100)
Motorcycle	80 (63.0)	41 (32.3)	6 (4.7)	127 (100)
Total	90 (49.2)	83 (45.4)	10 (5.4)	183 (100)

X^2^ = 45.2, *df* =2, *p*=0.006

**Table 3:** Pattern of skeletal injuries by gender and site of fracture

	mandible		midface	
Sex	male	130	male	52
	Female	29	female	22
Total		159		74
Site	mandible	f	midface	f
	Condyle	32	Le fort 1	21
	Ramus	4	Le fort 11	18
	Angle	28	Le fort 111	6
	Body	43	Zygoma	29
	Symphysis	24	nasal bones	5
	Dentoalveolar	41	Dentoalveolar	21
Total		172		100

**Table 4:** Altered consciousness level in relation to aetiology and fracture site

Aetiology/ site	Altered consciousness		Total
	Yes	No	
Motor vehicle	11	45	56
Motorcycle	80	47	127
Assault	2	44	46
Fall	0	2	2
Sport	0	2	2
Total	93	140	233
X^2^=61.6 *df*=4 *P*=0.001			
Site of fractures			
Mandible	39	120	159
Maxilla	54	20	74
Total	93	s 140	233

X^2^=49.6 *df*=1 *P*=0.003

**Table 5:** Concomitant injuries

Type of injury	Number (%) of patients
Craniocerebral	26 (60.5)
Cervical spine	8 (18.6)
Rib fracture	2 (4.7)
Upper limb	2 (4.7)
Lower limb	3 (6.8)
Eye globe	2 (4.7)
Total	43 (100)

**Table 6:** Treatment of maxillofacial fractures

Mandible	Frequency
2.4mm titanium plates osteosynthesis	48
Eyelet wiring/ arch bars/ MMF	79
Transoosseous wiring	11
No active treatment	34
**Midface**	
***Maxilla***	
Miniplate osteosynthesis	17
Circumzygomatic- mandibular suspension + MMF	5
Fronto-mandibular wire suspension + MMF	3
Eyelet wiring/ arch bars/ MMF	15
No active treatment	26
***Zygomatic fractures***	
Elevation via Gillies temporal approach	6
Miniplate osteosynthesis via lateral eyebrow incision	4
Fronto-mandibular wire suspension + MMF	3
No active treatment	16
***Nasal fractures***	
Reduction with Walsham’s forcep	3
No active treatment	2
**Total number of fractures**	**272**

## Discussion

Trends and characteristics of maxillofacial injuries vary from one population to another depending on certain peculiarities such as socioeconomic, cultural and environmental factors [6, 7]. The Ekiti State University Teaching Hospital is a referral centre for Ekiti and four neighbouring states, serving a combined population of about ten million people. In addition, the state provides a major link for road transport between the North-central and south-western parts of Nigeria. Thus, we provide trauma care for a sizeable number of injured commuters along this route. The observed incidence in age is similar to previous reports where majority of the patients were in the third decade of life [1, 8]. The third decade marked the active phase whereby individuals are more likely to indulge in injury prone adventures such as dangerous driving, violence and use of automobile for commercial purposes. Also, the advent of the use of motorcycle for commercial purpose in most Nigerian cities has been linked to the rising spate of youth unemployment [7, 25]. The male preponderance of more than twice the number of females is also in agreement with previous reports where majority of injured patients were male [1, 6-9] RTC was the commonest aetiology of fractures in this study with a frequency of 78.5%. This is higher than the value reported by Adebayo et al [1] (56%) and Cheema and Amin [5] (56%) but comparable to the study by Adekeye et al [21] (76%), Ugboko et al [9] (72%) and Fasola et al [11] (71%), However, Oginni et al [7] and Abiose et al [22] reported slightly higher prevalence of 81.6 and 81% respectively. The fact that motorcycle related fractures constitute majority of RTC corroborates findings from similar studies [7, 19, 26]. In agreement with earlier reports, other causes of maxillofacial fractures such as assault, falls and sports were low in prevalence in this study [1, 9, 11]. The advent of motorcycles for transportation has a significant contribution to RTC in Nigeria because most riders are uneducated youths who do not adhere to traffic rules on protection and road usage [6, 7]. Rising youth unemployment and rural/ urban migration has further popularised the use of motorcycle for commercial purpose in Nigeria [7, 19, 26]. Furthermore, motorcycles can navigate narrow ways, bypass traffic jams and they often carry more than the recommended number of pillion passengers which predisposes the automobile to RTC. Also, airbags and other protective devices which are absent in the motorcycle further endanger the commuters to RTC. The majority of motorcycle related injuries involved the rider contrary to motor vehicle crashes where passengers were mostly involved (p=0.006). Motorcycle riders rarely wear helmet with protective visor or strapping as a low proportion of the patients had crash helmet on at the time of injury. Similarly motor vehicle passengers, especially on the back seat are seldom protected by airbags and they rarely use seatbelts. The poor state of Nigerian roads and consistent disregard for road safety measures by commuters may explain why RTC has overshadowed other causes of oral and maxillofacial fractures in our environment.

In agreement with some studies, the mandible was the most frequently fractured bone of the face in our series with the body being the most commonly affected part of the bone [7, 11, 14, 20]. However, Odusanya [27] in a previous study from Nigeria established that the condyle was the most commonly fractured part of the bone. Similarly, recent reports by Mesgarzadeh et al [14] in Iran and Al Ahmed et al [12] in UAE revealed higher prevalence of fracture of the condyle. Some researchers also corroborated higher frequency of condylar fractures in North America and Europe [17, 18]. However, findings from this study supported the claim of other authors who reported that fracture of the body and symphysis of the mandible has been observed to be related more to motocycle crashes [7, 19, 26]. In the current study, zygomatic bone was the most commonly fractured in the midface as alluded to by other studies [9, 11, 22, 28]. Midface fractures were reported to be less prevalent than mandibular fractures probably because of the mobility of the latter and its proneness to injury [2, 6, 7]. However, Gassner et al [29, 30] in two separate maxillofacial injury surveys reported a higher prevalence of midface fractures. A number of midface fractures are often missed because such cases are poorly demonstrated on plain radiograph which is the commonest imaging modality in most Nigerian health institutions. The CTscan which is the imaging of choice for maxillofacial fractures is restricted to use only in patients who can afford the high cost of such technique. Regarding altered state of consciousness level; anecdotal report has shown that a significant number of patients with maxillofacial injuries often suffer head injury with attendant loss of consciousness. However, there is dearth of published report on concomitant injuries in most maxillofacial trauma surveys generally. In the present study, the proportion of those who had loss of consciousness was similar to the findings of Oginni et al [7] [43%]. Similarly the relationship between the cause of injury and altered state of consciousness was statistically significant (p=0.001). However, contrary to the study by Oginni et al, there was a statistically significant association between fracture site [mandible or midface] and altered consciousness in this study (p=0.003). This finding was supported by Zhan et al [31] who stated that occurrence of head injury had significant relationship with aetiology and site of fracture. We opine that the closeness of the bones of the midface and the articulations with the skull base may be responsible for the high incidence of head injury observed in patients with midface fractures. Craniocerebral injury (60.5%) which was the commonest concomitant injury in our series was also reported by some authors as the most common associated injury [9, 32, 33]. The incidence of head injury can be linked to the impact of collision especially where protective devices like helmet and seatbelts are not in use at the time of injury. Although open reduction and internal fixation was employed for fewer proportion of fractures treated in this study, satisfactory result was obtained with minimal complications. Until the advent of osteosynthesis, MMF and wire suspension were the treatment of choice for facial fractures in Nigeria [1, 5, 8, 29]. MMF and wire suspension are still largely popular because a lot of Nigerians are unable to afford the cost of bone plates.

## Conclusion

Road traffic crashes remain the major cause of maxillofacial injuries and motorcycle related crashes were predominantly responsible for most fractures. These injuries are often associated with concomitant injuries and attendant loss of consciousness. Thus there is need for continual advocacy on preventive measures and enforcement of traffic rules to reduce the observed high incidence of maxillofacial fractures.

### What is known about this topic

- Road traffic crashes is the commonest cause of maxillofacial fractures in developing countries, Nigeria inclusive;
- There is high male preponderance and youths are mostly affected;
- There is still controversy in the literature regarding the commonest site of fracture on either the mandible or the midface.

### What this study adds

- Pattern of presentation of patients with facial bone fracture in our institution;
- prevalence of associated head injury and attendant loss of consciousness;
- The status of the patient and site of fracture [either mandible or midface] has a statistically significant relationship with loss of consciousness. Also, Motorcycle related crashes have a statistically significant relationship with loss of consciousness compared to the other agents of injury.
